# The Army Medical Corps

**Published:** 1901-04-27

**Authors:** 


### The Hospital

A Journal of

The Medical Sciences and Hospital Administration.

Vol. XXX.—No. 761] Edited by Sir Henry Burdett, K.C.B., and Solomon C. Smith, M.D., M.R.C.P. [April 27, 1901.

### THE ARMY MEDICAL CORPS.

Whatever substratum of truth may have existed for the aspersions which, at one period of the war in South Africa, were somewhat liberally cast upon the general medical arrangements, the publication, last week, of Lord Roberts's maturely considered despatches will suffice to show that in the medical and nursing departments, as in all others, the Army lately under his command has been true to the great traditions of the British military service, and has thoroughly established a claim to the gratitude of the nation. "Some cases," writes Lord Roberts, "have been brought under my notice in which officers have proved unequal to the exceptional strain thrown upon them by the sudden expansion of hospitals; and, in the earlier stages of the war, the necessity of more ample preparations to meet disease was not quite fully apprehended." In every large body of men, individual cases of inadequacy to meet exceptional strain must of necessity be encountered; and may often, no doubt, be due to temporary causes affecting the individuals concerned, to such causes as indisposition not amounting to disabling illness, or to loss of rest, or to insufficient food; and such occurrences cannot be held in any way to detract from the general merits of the body concerned. With regard to the "necessity of more ample preparations," Lord Roberts hastens to put the saddle upon the right horse, by indicating this as a matter which will receive the attention, not of the officers of the corps, but "of His Majesty's Government." He sufficiently emphasises what was already perfectly well known, namely, that the shortcomings with regard to hospitals which were only too notorious at one period of the war were in no way to be attributed to the medical department, but solely to the inability of the War Office to recognise the probable magnitude of the demands which would arise, and to provide for them in a manner commensurate with their urgency and their importance. Even in this matter, it is easy to be wise after the event; and the fairest criticism will be based upon a frank admission that not only the War Office, but the Army and the nation, fell into the not unnatural error of underrating the resisting powers of the adversary, and of underestimating the difficulties of the campaign. It was not alone in doctors and in hospital equipment that our provisions fell far short of those which events proved to be required. The considerations hence arising will, we hope, be borne in mind whenever the nation is again engaged in serious conflict; but for the present, at least, they may be dismissed as irrelevant to any questions now before us; and we may turn from them to note the testimony of Lord Roberts to the merits of the officers whose conduct and efficiency were at one time seriously impugned, nothwithstanding the verbal disclaimers of any desire to cast personal blame upon them which were profusely made by the accusers. Lord Roberts goes on to speak of the "unremitting services" of the great majority of the officers of the Royal Army Medical Corps, and of the many instances of great gallantry displayed by them in carrying on their work of mercy under heavy fire. Nor is he less emphatic in his recognition of the excellent work done by the distinguished consulting surgeons who proceeded to South Africa, and by their advice and experience rendered material assistance. But, after all, his highest eulogium is reserved for the nursing sisters, with regard to whom he finds it difficult, within the limits of a paragraph, to give expression to the "deep feeling of gratitude" with which they have inspired all ranks. He speaks of the "devotion, skill, courage, and endurance," displayed equally by the Army Nursing Service and by kindred organisations from the Colonies; and adds that some of the most helpful of the nurses have been lent to the Army Nursing Reserve by the great hospitals in the United Kingdom. In a future despatch he proposes to mention the names of some of the most deserving; and we feel sure that nothing would afford greater gratification, either to the Army or to the public, than to see the merits of these ladies recognised by the bestowal upon them of honours which should serve as lasting testimonies of their merit. The extent to which this has been already done in the cases of civilian surgeons will be extremely satisfactory to the profession, and marks an enormous advance in public and in official opinion since the days of the Crimea. The civilian surgeons who then responded to the call of the War Office for assistance received only a scanty stipend, and no other recogni tion of the work they had done or of the dangers they had incurred than the medal which was given to every private soldier. The Turkish Government wished to confer the decoration of the Medjidieh upon the English civil surgeons who accompanied their army into Mingrelia and took part in the battle on the Ingour, but the War Office refused the necessary permission. On this occasion, the publication of Lord Roberts's despatch is immediately followed by the gratifying announcement that Sir William MacCormac adds the K.C.B. to his other well-earned distinctions; and that the Companionship of the same Order has been conferred upon Sir Thomas Fitzgerald, Sir William Thomson, Messrs. Fripp, Makins, Treves, Watson Cheyne, and Cheatle, and Drs. Franks and Chiene. The names of no less than sixty-one officers of the Army Medical Corps are mentioned by Lord Roberts as deserving of notice for meritorious services performed; and eleven of these have already received promotion. It is noteworthy that to no less than three out of the eleven, namely, Lieutenant-Colonel Baptie, Captain Nickerson, and Captain Inkson, the Victoria Cross had been previously awarded in recognition of distinguished bravery during the campaign.

